# Supposed Case of Ovarian Hernia, Forming an Ovarian Tumor

**Published:** 1847-11

**Authors:** F. H. Hamilton

**Affiliations:** Buffalo


ART. III.—*Supposed case of Ovarian Hernia, forming an Ovarian Tumor.* Reported by Dr. F. H. HAMILTON.

Miss M. E., aged 21, of Buffalo, has a tumor situated upon the external abdominal ring of the right side, of which the history is as follows:—

When two weeks old, she was lying upon the bed with her face upward, when her sister, ten years old, accidently jumped upon her chest and abdomen, with such force as to nearly extinguish the life of the infant, bending and flattening the ribs upon one side, and forcing out the sternum. During the succeeding week she was very feeble, and was apparently in much pain when moved. About this time the mother discovered a small tumor situated on the external abdominal ring, of the size and shape of a kidney bean, but thicker; not discolored, and moveable: it could not be returned within the ring. This tumor has never disappeared for one moment since then.

Miss M. began to menstruate when 13 years old, and from the time of the accident unntil this event, the groin gave her much trouble. When she played much, a swelling would occur *beside* the tumor, (the small original tumor remaining distinct and clearly separate) often attaining the size of a hen's egg, giving her great pain until it was reduced, never red, but sometimes purplish, and always tender. This swelling was easily made to disappear in from half an hour to three hours, by applying warm cloths, and keeping her on her back; sometimes it disappeared spontaneously, while she still kept about. It would "swell up" as often as three times a week on the average, except during two years—the third third and fourth year of her life. The disappearance of the swelling at this time, the mother ascribes to her having used "fox grease" upon it.

Her menstruation has always been painful, scanty, and irregular; she has once, passed five months without menstruating. But whether she menstruates or not, the periodical day is generally announced by some tumefaction and pain in the original tumor, often a very severe pain; the pain occasionally making a sudden metastasis to the loins, or hips, or perhaps, the head. The pain and tumefaction, also, regularly subside after two or three days, although she may not have menstruated. She has a constant blenorrhagia from the vagina, and prolapsus of the uterus. When 15 years old she was seized with a sudden and unusually severe pain in the tumor, which lasted two or three days, and was relieved after an abrupt and copious discharge of offensive matter through the vagina.

Shortly after this event I first saw the patient, in consultation with Dr. Rhodes, then near Geneva, Jan. 1842. She was exceedingly feeble, and the original tumor (not the occasional tumor, which I designated as a "swelling") had within a few days attained the size of a large goose egg. A careful examination enabled us to determine that it was neither a hernia nor a lumbar abscess, to both of which it presented several points of resemblance. It fluctuated distinctly, and was exqusitely tender, but could not be made to retire within the ring. Dr. Rhodes opened it freely; it discharged a pale yellow serum. The probe introduced discovered an arrangement of the interior by septa into copartments, and a distinct and firm cyst involving the whole.

Three or four years since, it again enlarged in the same manner, and having attained about the same size, it was opened freely by Dr. Briggs, of Auburn. The matter discharged possessed the same character as before.

On the 9th inst., I was called to her again, she having now removed to this city. Her general health has somewhat improved since I saw her in 1842, but she has still vaginal blenorrhagia and prolapsus; she has also a polypus in each nostril; palpitation of heart; and has had, she now tells me, enlargements of the glands of the neck, and also in both groins.

The tumor has now swelled again for the third time, and, as has always been the case before it is opened, it is intensely painful, and she is compelled to seek her bed.

I opened it, and it discharged about two ounces of a pale yellow serum. A tent is left in the wound.

I have occupied so much time in the description of this case, because I believe it to be an *ovarian hernia*, a rare, yet possible accident, which has now become diseased, and has formed in this new bed a true *ovarian tumor*. The *occasional* swelling, which recured so often previous to puberty, although the mother thinks she never could reduce it by pressure, I believe to have been an intestinal hernia, which easily found its way through the track made by the ovary. The intestinal hernia was, however, prevented from decending when the ovary itself took on disease, which by enlargement and inflammation blocked up the channel.

The girl will not consent to its extirpation. I have omitted to say that the deformity of her thorax still remains.

BUFFALO, October, 1847.

